# Combined Metabolome and Transcriptome Analyses Reveal the Flavonoids Changes and Biosynthesis Mechanisms in Different Organs of *Hibiseu manihot* L.

**DOI:** 10.3389/fpls.2022.817378

**Published:** 2022-03-15

**Authors:** Yuhan Zhou, Xiaodi Xu, Yanzhu Chen, Jun Gao, Qiyu Shi, Liang Tian, Li Cao

**Affiliations:** Agricultural College of Yanbian University, Yanji, China

**Keywords:** LC-MS/MS, RNA-seq, flavonoid, *Hibiseu manihot* L., flavone, flavonol

## Abstract

*Hibiseu manihot* L. (Jinhuakui, JHK), also known as a garden landscape plant, is widely cultivated as a landscape plant having pharmacological effects due to its high flavonoids content. Although flavonoids were the main active pharmaceutical ingredients in JHK, little information was obtained about the content, composition, and accumulation pattern of flavonoids in different tissues. Most studies only identified a few kinds of flavonoids in JHK limited by separation and identification problems. Therefore, combined metabolome and transcriptome analysis was performed to explore the accumulation patterns and biosynthesis mechanisms of flavonoids in JHK. In this study, we identified 160 flavonoids in 15 samples of JHK (flower, leaf, root, stem, and seeds) by using LC-MS/MS. Consistent with the total flavonoid content determination, these flavonoids were significantly accumulated in flowers, followed by leaves, stems, roots, and seeds. Among them, certain flavonoids, with high content, were also identified for the first time in JHK, such as tricetin, catechin, hesperidin, ncyanidin-3-O-sambubioside, astragalin, procyanidin B2/B3/C1, apigenin-5-O-glucoside, etc. Different tissues underwent significantly reprogramming of their transcriptomes and metabolites changes in JHK, particularly in the flavonoid, flavone, and flavonol biosynthesis pathways. We conducted a correlation analysis between RNA-seq and LC-MS/MS to identify the key genes and related flavonoids compounds, rebuild the gene-metabolites regulatory subnetworks, and then identified 15 key genes highly related to flavonoids accumulation in JHK. These key genes might play a fine regulatory role in flavonoids biosynthesis by affecting the gene expression level in different organs of JHK. Our results could be helpful for the improvement of the market/industrial utilization value of different parts of JHK, to pave the way for the regulatory mechanism research of flavonoids biosynthesis, and provide insight for studying the production quality improvement of JHK.

## Introduction

*Hibiseu manihot* L., one of the endangered plants from the family of Malvaceae and the genus of *Abelmoschus*, is recorded as “Jinhuakui” (JHK) in Chinese, and distributed in the Hebei province of China (Cao and Miao, 2016). The plant shape of JHK is like okra, with large flowers and golden corolla, which can be eaten directly, and has high ornamental value and nutritional value among more than 200 okra plants (Liu, 2008; Peng and Wu, 2008). Therefore, JHK can be used for the development of landscape plants, medicinal plants, and healthy food. Previous studies have shown that JHK has high medicinal value, and the whole plant, including flowers, leaves, roots, stems, and seeds, exhibit wide pharmacological activities (Peng and Wu, 2008; Lan et al., 2012) and can be used as medicine in China. The flowers, fruits, and seeds of JHK have multiple pharmacological effects, which are effective in relieving pain and reducing inflammation as well as applied it to treat traumatic injuries or sprains (Chen L.M. et al., 2016). Pharmacological research studies have shown that the extract of JHK has been used in clinical practice as a remedy for certain diseases, and it also possesses a good therapeutic effect in antioxidation, anticonvulsant, antiinflammation, and immune regulation (Lu and Jia, 2015). The extracts of JHK (flowers, leaves, stems, roots, and seeds) have potential biological active constituents that are responsible for inhibition of inflammation effects or wound healing bioactivity (Zhang et al., 2019). Therefore, studying the compounds (active ingredients or secondary metabolites) of JHK could serve as potential resources for the development of health products that possessed bioactive properties.

Previous research reported that the JHK was rich in lots of bioactive components, such as flavonoids, organic acids, volatile oil, aliphatic hydrocarbons, polysaccharides, trace elements, vitamin E, etc. (Li et al., 2012; Chen L. et al., 2016; Chen L.M. et al., 2016). Among them, flavonoids are the main bioactive ingredients in *Hibiscus Manihot* L. (JHK) (Xia, 2007). The flower of JHK has the highest total flavonoids content compared with other tissues, which is up to 8.47%, followed by leaves, seeds, stems, and roots (Wei et al., 2012; Lu and Jia, 2015; Wang, 2015; Song et al., 2016). The content of total flavonoids accumulates in JHK was ten times higher than in other flavonoids rich plant species (raw material) that are widely used to extract flavonoids in industry, such as ginkgo and soybeans (Wei et al., 2012; Lu and Jia, 2015). Thus, JHK can be used as one of the raw materials for the extraction of total flavonoids. Studies have reported that JHK is rich in hyperin, rutin, quercetin, myricetin, and other active flavonoid monomers, which have pharmacological activities to alleviate inflammatory, and convulsant (Xia, 2007; Zhang et al., 2019). Systematically studying the chemically active components, classification of flavonoids and the mechanism of flavonoids synthesis in JHK have great significance for human health.

Until now, the previous research on the JHK was mainly focused on the optimization of the cultivation mode (Fan et al., 2020), exploring seed germination conditions (Yu, 2021), optimization of extraction method of total flavonoids in JHK (Cui et al., 2020), and a limited number of flavonoids and polyphenol identification (Tsumbu et al., 2012; Wang M. et al., 2021), such as hyperoside, caffeic acid, chlorogenic acid, rosmarinic acid, rutin, and tannins. Although there are certain studies on flavonoids in JHK, most of them only studied its extraction, detection, and preliminary activity analysis. Most of the studies are mainly on the phytochemical constituents and biological activities of the relative species of *Abelmoschus manihot* (Huangshukui in Chinese) (Lai et al., 2009; Pan X. et al., 2017). However, limited information on the metabolic compound (chemical constituents) and biological activities, especially the types of flavonoids in JHK still remained unknown. It is known that the vegetation stage of plants together with environmental conditions may affect changes and the formation of phenolic and flavonoids compounds (Sytar et al., 2013, 2014; Li et al., 2020). Furthermore, the dynamic change and distribution differences of these flavonoids in the flowers, leaves, roots, stems, and seeds of JHK were also unclear.

In recent years, metabolomics combined with transcriptomics (RNA-seq) provides a powerful tool for the investigation and characterization of postgenomic processes and the molecular basis in many plants (Morgenthal et al., 2006). The metabolomics and RNA-seq profiles have been analyzed in many plants to expose the mechanism of metabolic compounds synthesis and dynamic changes in different plant species, including *Abelmoschus manihot* (Pan X. et al., 2017), tea plants (Wang S. et al., 2021), jujube (Li S. et al., 2021), passion fruit (Li C. et al., 2021), *Sophora alopecuroides* (Zhu et al., 2021), longan (Yi et al., 2021), etc. Therefore, to systematically study the types, distribution, and dynamic changes of flavonoids in different tissues of JHK, in the present study, metabolomics combined with transcriptome analysis was performed in different tissues (flowers, leaves, stems, roots, and seeds) of JHK. The distribution and content ratio of flavonoids in different tissue of JHK were systematically studied and compared, and the mechanism of flavonoid synthesis and related key genes were also identified. This study will pave the way for the research on the active constituents and pharmacology of JHK, and provide the basis and reference for further study on the mechanism of flavonoids synthesis.

## Materials and Methods

### Plant Materials and Treatments

**FIGURE 1:**
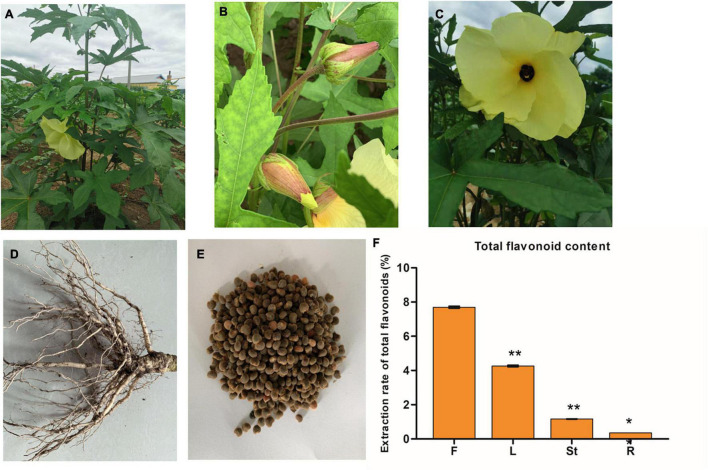
The phenotypes of the whole plant **(A)**, leaves **(B)**, flower **(C)**, roots **(D)**, and seeds **(E)** of *Hibiseu manihot* L.; and the total flavonoid content in different tissues of *Hibiseu manihot* L. **(F)**, i.e., flower (F), leaves (L), stems (St), and roots (R). The asterisks (** and *) above each bar represent a significant difference at *p* < 0.01 and *p* < 0.05, respectively.

The flowers, leaves, stems, roots, and seeds of the JHK were used in this study (Figure 1). All flowers, leaves, stems, roots, and seeds samples were collected from the Shimen Town, Antu County, Yanbian Korean Autonomous Prefecture, Jilin Province (129.02°E, 43.03°N), which were planted at the end of May 2020 and sampling was done at the end of August 2020. The average annual temperature is around 3–5°C, with the coldest January averaging minus 18°C and the hottest July averaging around 20°C. The flowers, leaves, stems, roots, and seeds of JHK were named F, L, St, R, and Se, respectively. Five plants with the same (or close) growth were selected as one repeat, with a total of three biological replicates in each group. The flowers, leaves, stems, roots, and seeds of the JHK were sampled separately, and the soil at the roots was rinsed repeatedly with distilled water. The surfaces of leaves, seeds, and flowers of JHK were also rinsed with distilled water. All these samples were quickly frozen in liquid nitrogen containers and brought back to the laboratory, and stored at −80°C refrigerators for further experimental analysis.

### Determination of Total Flavonoids Content

The tissues of JHK were ground in liquid nitrogen using a high-speed multifunction pulverizer (Zhejiang, China). The sample powder (5.0 g) was weighed and extracted by ultrasonic method (40 kHz) under the conditions of ethanol concentration of 80%, extraction time of 30 min, extraction temperature of 60°C, and the solid-liquid ratio of 1:20 g/ml. The filtrate was then centrifuged and transferred to a 50 ml volumetric flask after rotary evaporation by RE 52-99 rotary evaporator (Shanghai, China). Then, 80% ethanol solution was used for constant volume, and the filtrate was shaken well for reserve use. The sampling solution was used as the blank control and the experimental group (flowers, leaves, stems, roots, and seeds) was taken in colorimetric tubes, and then 80% ethanol solution was added to 5.0 ml successively. Then 5.0 ml of 80% ethanol solution was added to the sample calibration tube to make the volume constant at 10 ml. Blank control and sample solution were added to 0.3 ml 5% Na_2_NO_2_ solution, shaken well, and left for 6 min. Then 0.3 ml was added to 10% Al (NO_3_)_3_ solution, shaken well, and left for 6 min. Then 4.0 ml of 1 mol/L NaOH solution, and 0.4 ml deionized water were added, shaken well, and left to stand for 15 min. The absorbance (OD) was determined by spectrophotometer at 510 nm (7230G Visible Spectrophotometer, Shanghai, China), and the content of total flavonoids was calculated by standard curve. Rutin was used as a reference standard, and results were expressed as rutin equivalents in mg/kg extract.


Extractionrate(%)=(flavonoidcontentintheextract/weightofthesampleused)×100%

### Sample Preparation for UPLC-MS/MS

The sample was placed in a lyophilized machine (SCIENTZ-100F) for vacuum freeze-drying. Then, the freeze-dried samples were ground to a powder using a mixer mill (MM 400, Retsch) with a zirconia bead for 1.5 min at 30 Hz. A total of 100 mg of powder was weighed and dissolved in 1.2 ml 70% aqueous methanol (v/v) for extraction. The samples were vortexed every 30 min for 30 s for 6 total vortexes and placed in a refrigerator overnight at 4°C. Then, the extracted samples were centrifuged (10,000 *g*, 10 min), absorbing supernatant for filtration by Millipore filtration system (0.22 μm pore size, ANPEL, Shanghai, China) and stored in the injection bottle for UPLC-MS/MS analysis.

### Metabolite Profiling by UPLC-MS/MS

The metabolite data acquisition instrument system mainly includes ultraperformance liquid chromatography, UPLC (SHIMADZU Nexera X2^1^) and tandem mass spectrometry, MS/MS (Applied Biosystems 4500 QTRAP^2^). Liquid phase conditions were as follows: HPLC column, Agilent SB-C18 1.8 μm, 2.1 mm × 100 mm; solvent system, phase A is ultrapure water (adding 0.1% formic acid), phase B is acetonitrile (adding 0.1% formic acid); gradient program, phase B is 95:5 V/V at 0.00 min, 5:95 V/V at 10.0 min, 5:95 V/V at 11.0 min, 95:5 V/V at 12.1 min, 95:5 V/V at 14.0 min; flow rate, 0.35 ml/min; temperature, 40°C; injection volume: 4 μl.

The mass spectrometry analysis was followed based on the method of Li S. et al. (2021) with slight modifications. LIT and triple quadpole (QQQ) scans were obtained on a triple quadpole linear ion trap mass spectrometer (Q Trap), AB4500 Q Trap UPLC/MS/MS system equipped with an ESI turbo ion-spray interface. It can be controlled by analyst 1.6.3 software (AB SCIEX) to run both positive and negative ion modes. The ESI source operation parameters are as the follows: ion source turbo spray; source temperature 550°C; ion spray voltage (IS) 5,500 V (Positive ion mode)/−4,500 V (negative ion mode); ion source gas I (GSI), gas II (GSII), and curtain gas (CUR) set at 50, 60, and 25.0 psi, respectively; and high collision gas (CAD). The instrument was tuned and calibrated with 10 and 100 μmol/L polypropylene glycol solution in QQQ and LIT modes, respectively. The QQQ scans were acquired as multiple reaction monitoring (MRM) experiments with the collision gas (nitrogen) set to medium. Through further optimization of DP and CE, the DP and CE of each MRM ion pair were completed. A specific set of MRM ion pairs was monitored in each period based on the metabolites eluted in each period.

### Qualitative and Quantitative Analyses of Metabolites

Based on the self-built MWDB databases (Metware Biotech, Wuhan, China^3^) and public metabolite databases, ChemBank^4^ ; PubChem,^5^ MassBank,^6^ NIST Chemistry Webbook,^7^ KNAPSAcK,^8^ MoToDB,^9^ HMDB,^10^ and METLIN,^11^ the metabolites were characterized according to the second-order spectral information. During the analysis, isotope signals, repeated signals containing K+ ions, Na+ ions, NH4+ ions, and fragments of other substances with larger molecular weight were removed. MRM analysis of QQQ mass spectrometry was performed to quantitative analysis of metabolites. In the MRM mode, the quadrupole firstly screened the precursor ions (parent ions) of the target substance and excluded the related ions of other molecular weight metabolites to preliminarily eliminate the interference. After induced ionization in the collision chamber, the precursor ions were fractured to form a lot of fragments, and then the fragments were filtered through the triple four-bar filter to select a characteristic fragment ion needed to eliminate the interference of non-target ions so that the quantification is more accurate and the reproducibility is better. Finally, the mass spectrum peak area was used to determine the relative metabolite contents (Fraga et al., 2010).

The filtered metabolite data were analyzed by Analyst 1.6.1 software for orthogonal partial least squares-discriminant analysis (OPLS-DA) and unsupervised principal component analysis (PCA). The relative importance of each metabolite to the OPLS-DA model was checked using the parameter called variable importance in projection (VIP). Hierarchical clustering analysis of the metabolites between each sample was performed using R software.^12^ The screening criteria, | log2(fold change) | ≥ 1 and VIP ≥ 1, was used to identify differentially accumulated metabolites (DAMs) in this study.

### RNA-Seq Analysis

The total RNA of JHK (F, L, St, R, and Se) was extracted using the FastPure plant total RNA isolation kit (Vazyme, RC401) according to the manufacturer’s protocol. Fifteen sequencing libraries were generated using NEBNext Ultra RNA Library Prep Kit for Illumina (NEB, United States) following the manufacturer’s recommendations and index codes were added to attribute sequences of each sample. Divalent cations under elevated temperature in NEBNext First Strand Synthesis Reaction Buffer (5×) were used for fragmentation. Random hexamer primer and M-MuLV reverse transcriptase were used to synthesize first-strand cDNA. Second-strand cDNA synthesis was subsequently carried out using DNA Polymerase I and RNase H. TTo select cDNA fragments of preferentially 240 bp in length, the library fragments were purified with the AMPure XP system (Beckman Coulter, Beverly, MA, United States). The clustering of the index-coded samples was performed on a cBot Cluster Generation System using TruSeq PE Cluster Kit v3-cBot-HS (Illumia) according to the manufacturer’s instructions. After cluster generation, the library preparations were sequenced on an Illumina Hiseq 2000 platform, and the paired-end reads were generated. The sequences were further processed with a bioinformatic pipeline tool, BMKCloud^13^ online platform. Raw data (raw reads) of fastq format were firstly processed through in-house perl scripts. After removing reads containing adapter, reads containing ploy-N and low-quality reads from raw data and clean data (clean reads) were obtained. Clean reads were assembled into expressed sequence tag clusters (contigs) and *de novo* assembled into the transcript by using Trinity. At the same time, Q20, Q30, GC-content, and sequence duplication levels were calculated in the clean data. All the downstream analyses were based on clean data with high quality. The function of the genes was annotated based on the databases, namely, NR (NCBI non-redundant protein sequences), KOG/eggNOG (clusters of orthologous groups of proteins), Pfam (protein family), SwissProt (a manually annotated and reviewed protein sequence database), GO (gene ontology), KEGG (Kyoto Encyclopedia of Genes and Genomes). Differentially expressed genes (DEGs) were identified using the DESeq functions estimate size factors and the nbinom test. A value of *p* < 0.05 and fold change > 2 or fold change < 0.5 was set as the threshold for significantly differential expression.

### Quantitative Real-Time PCR Analysis

Twenty DEGs in the flavonoid synthesis pathway were selected for quantitative real-time PCR (qRT-PCR) analysis. The primers were designed using Primer 3^14^ and they are listed in Supplementary Table 1. The RNA was extracted from JHK and was used to synthesize first-strand cDNA by using TransScript First-Strand cDNA Synthesis SuperMix (TransGene, AT301-02) following the manufacturer’s instructions. qRT-PCR was performed using the SYBR Green PCR kit (Qiagen, 204054) according to the manufacturer’s instructions. In this study, all the genes were repeated in three biological replicates (each biological replicate contains 3 technical replicates). The 2^–ΔΔ*Ct*^ method was used to calculate the mRNA expression level of genes. The relative gene expression level and FPKM were normalized by using log2 (fold change) measurements. The R software package version 3.1.3^15^ was performed to analyze the correlation between RNA-seq and qRT-PCR data.

## Results

### Determination of Total Flavonoids Content in *Hibiseu manihot* L.

Flavonoids are the main metabolites and medicinal ingredients in the JHK plant. Therefore, the total flavonoids content in different tissues (flowers, leaves, stems, and roots) of JHK was firstly detected in this experiment. The results showed that the content of total flavonoids in flowers was significantly higher than that in leaves, stems, and roots (*p* < 0.01). Among them, the total flavonoids content in flowers of JHK was 44.46% higher than that in leaves, while the total flavonoids content in roots and stems was very low (Figure 1F). Therefore, to explore the mechanism of the changes in flavonoid accumulation in different tissues of JHK, the flowers, leaves, stems, roots, and seeds of JHK were studied in the present experiment by using LC-MS/MS and RNA-seq.

### Principal Component Analysis and Sample Correlation Analysis of *Hibiseu manihot* L.

Firstly, we carried out a quality control (QC) analysis on the metabolite detection results of these samples. The superposition diagram of the total ion chromatogram (TIC) detected by the QC sample essential spectrum is shown in Supplementary Figure 1. The results showed that the curves of the total ion flow detected by the metabolites had a high overlap in JHK, that is, the retention time and peak intensity were consistent, which indicated that the signal was stable when the same sample was detected at different times by mass spectrometry. The high stability of the instrument provides an important guarantee for the repeatability and reliability of the data, where N represents the negative ion mode and P represents the positive ion mode. Therefore, the results indicated that the metabolite detection in this study is reliable and can be used for subsequent analysis.

**FIGURE 2:**
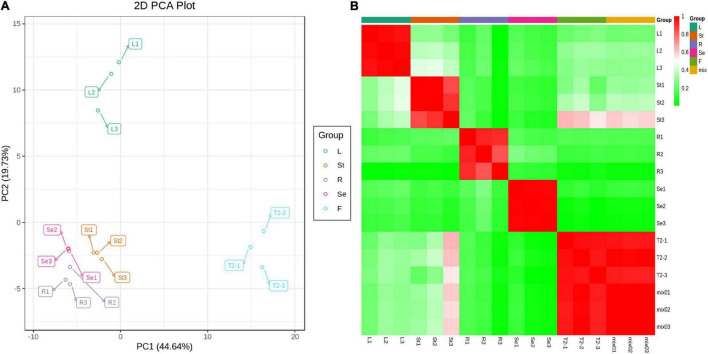
Principal component analysis (PCA) and sample correlation analysis in different tissues of *Hibiseu manihot* L. Metabolite PCA from the LC-MS/MS of each tissue group **(A)** and sample to sample clustering heat map of different tissues **(B)**. In LC-MS/MS profile analysis, F, L, St, R, and Se represent the flower, leaves, stems, roots, and seeds of *Hibiseu manihot* L., respectively.

Principal component analysis showed that the first principal component (PC1) could explain 44.64% of the total variance and distinguish samples based on the different tissues/organs of JHK (Figure 2A). The flower (T2 or F) was separated from the leaf (L), stem (St), seed (Se), and root (R) of JHK. There was a significant difference between the flavonoids in flowers and other tissue parts, which was consistent with the results of total flavonoids detection in the front section (Figure 1F). The second principal component (PC2) could explain 19.73% of the total variance and separate organs of L from others (F, St, Se, and R) in JHK (Figure 2A). It can be seen from PC2 that the flavonoids in the leaves of JHK were also significantly different from those in other tissue parts. The results of this experiment preliminarily concluded that the differences of flavonoids in flowers and leaves of JHK were significant compared with other tissue parts, while the differences of flavonoids in roots, stems, and seeds were not particularly significant.

The heat map of sample correlations (sample to sample clustering) showed that metabolites accumulation values among 15 samples of JHK were reproducible between the three biological replicates and batch effects were controlled (Figure 2B). Pearson’s correlation coefficient *r* was used as the evaluation index of biological repeat correlation. Pearson correlation coefficient is calculated by using the built-in cor function of R software. The *r*^2^ is closer to 1, the stronger correlation between the two repeated samples. At the same time, the higher the correlation coefficient between the samples within groups and the samples in different groups, the more reliable the differential metabolites were obtained. The present results showed that the reproducibility of different tissue parts of JHK was good and the experimental results were reliable.

### Analysis of Metabolites of Flavonoids in *Hibiseu manihot* L.

**FIGURE 3:**
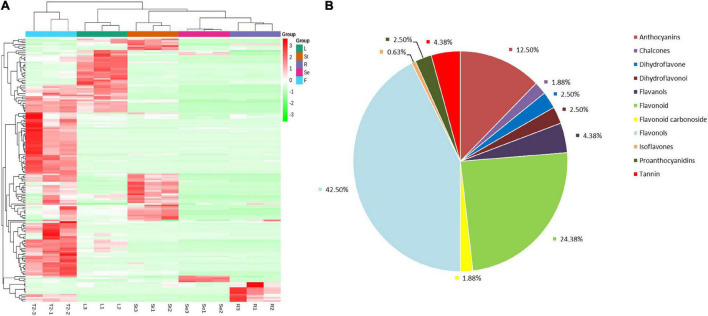
Overview of the flavonoids/metabolome profiles in different tissues of *Hibiseu manihot* L. Classification and proportion of all of the flavonoids into major functional class **(A)**; the total flavonoids/metabolite heat map derived from LC-MS/MS (non-targeted metabolome) profiling **(B)**. In the above heat map, the red color indicates significant flavonoids accumulation, and the green color indicates flavonoids content significant reduction. In LC-MS/MS profile analysis, F, L, St, R, and Se represent the flower, leaves, stems, roots, and seeds of *Hibiseu manihot* L., respectively.

A total of 160 flavonoids were detected by LC-MS/MS in JHK (Additional file 1). According to hierarchical cluster analysis (heat map Figure 3A), seed (Se) and root (R) were clustered into one cluster firstly, then cluster with stem (St) and leaf (L), while R, St, L, and Se were all clustered in different branches from the flower (F or T2) of JHK. The distribution and relative content of flavonoids in these tissues were also significantly different. It can be preliminarily seen from Figure 3B that the content of flavonoids in flowers is significantly higher than that in other tissues, followed by leaves and stems. In addition, the flowers, leaves, stems, seeds, and roots of JHK contained their own unique and significantly accumulated flavonoids (Table 1). Therefore, we conducted a detailed analysis of these 160 flavonoids identified in the present study. First of all, we classified the 160 flavonoids (Figure 3B), and these flavonoids mainly included anthocyanins (12.50%), chalcones (1.88%), dihydroflavone (2.50%), dihydroflavonol (2.50%), flavanols (4.38%), flavonoid (24.38%), flavonoid carbonoside (1.88%), flavonols (42.50%), isoflavones (0.63%), proanthocyanidins (2.50%), and tannin (4.38%). The results showed that these flavonoids may constitute the main pharmacological activity fraction of JHK. Among them, the content and types of flavonoids in flowers were the highest than other tissues.

**TABLE 1:** Classification and specific information of unique and high content flavonoids in various tissues and organs of *Hibiseu manihot* L.

Index	Q1 (Da)	Q3 (Da)	Molecular weight (Da)	Formula	Ionization model	Compounds	Class	CAS
**F**								
pme1598	463	301	464	C_22_H_24_O_11_	[M − H]−	Hesperetin 5-O-glucoside	Dihydroflavonol	69651-80-5
mws1361	449	151	450	C_21_H_22_O_11_	[M − H]−	Astilbin	Dihydroflavonol	29838-67-3
pmb0545	477	315	477	C_23_H_25_O_11_ +	[M]+	Rosinidin-3-O-glucoside	Anthocyanins	–
Zmcp002924	611	287	611	C_27_H_31_O_16_ +	[M]+	Cyanidin-3-O-(2′′-O-glucosyl) glucoside	Anthocyanins	38820-68-7
pme1773	595	287	595	C_27_H_31_O_15_ +	[M]+	Cyanidin-3-O-rutinoside (keracyanin)	Anthocyanins	28338-59-2
Smsp001641	625	317	625	C_28_H_33_O_16_ +	[M]+	Petunidin-3-O-rutinoside	Anthocyanins	–
Lmcp003402	479	317	478	C_22_H_22_O_12_	[M + H]+	Tamarixetin-3-O-glucoside (tamarixin)	Flavonoid	27542-39-8
Hmcp002187	509	347	508	C_23_H_24_O_13_	[M + H]+	Limocitrin 3-galactoside	Flavonoid	–
Zmhp005139	565	317	564	C_25_H_24_O_15_	[M + H]+	Tamarixetin-3-O-(6′′-malonyl) glucoside	Flavonoid	–
HJAP064	565	317	564	C_25_H_24_O_15_	[M + H]+	Isorhamnetin-3-O-(6′′-malonylglucoside)	Flavonoid	–
mws1002	345	330	346	C_17_H_14_O_8_	[M − H]−	Syringetin	Flavonoid	4423-37-4
mws2627	317	285	316	C_16_H_12_O_7_	[M + H]+	Tamarixetin (3,3′,5,7-tetrahydroxy-4′-methoxyflavone)	Flavonoid	603-61-2
Lmyp004617	419	257	418	C_21_H_22_O_9_	[M + H]+	Pinocembrin 7-O-β-D-glucoside (pinocembroside)	Flavonoid	75829-43-5
Hmlp003185	681	287	680	C_30_H_32_O_18_	[M + H]+	Luteolin-O-malonyl-O-hexoside-O-rhamnoside	Flavonoid	–
mws1003	331	151	332	C_16_H_12_O_8_	[M − H]−	Laricitrin	Flavonols	53472-37-0
Lmdp003808	317	302	316	C_16_H_12_O_7_	[M + H]+	Azaleatin (5-O-methylquercetin)	Flavonols	529-51-1
mws0856	463	301	464	C_21_H_20_O_12_	[M − H]−	Quercetin-4′-O-glucoside (spiraeoside)	Flavonols	20229-56-5
Hmgp001996	319	273	318	C_15_H_10_O_8_	[M + H]+	Quercetagetin	Flavonols	90-18-6
Lmmp002483	657	495	656	C_27_H_28_O_19_	[M + H]+	Gossypetin-3-O-glucuronide-8-O-glucoside	Flavonols	–
Lmmp003783	479	303	478	C_21_H_18_O_13_	[M + H]+	Quercetin-3-O-glucuronide	Flavonols	22688-79-5
pmn001640	449	316	450	C_20_H_18_O_12_	[M − H]−	Myricetin-3-O-arabinoside	Flavonols	132679-85-7
mws0855	317	139	318	C_15_H_10_O_8_	[M − H]−	Gossypetin (3,3′,4′,5,7,8-hexahydroxyflavone)	Flavonols	489-35-0
Lmmp002560	641	303	640	C_27_H_28_O_18_	[M + H]+	Quercetin-3-O-(2′′-O-glucosyl) glucuronide	Flavonols	–
Li512111	519	315	520	C_24_H_24_O_13_	[M − H]−	Isorhamnetin-3-O-(6′′-acetylglucoside)	Flavonols	–
Lmmp003767	463	287	462	C_21_H_18_O_12_	[M + H]+	Kaempferol-7-O-glucuronide	Flavonols	–
Hmcp001658	727	479	726	C_31_H_34_O_20_	[M + H]+	Isorhamnetin-O-hexoside-O-malonyl-O-Hexoside	Flavonols	–
Lmmp004257	463	317	462	C_22_H_22_O_11_	[M + H]+	6-C-methylquercetin-3-O-rhamnoside	Flavonols	–
Lmyp003349	617	153	616	C_28_H_24_O_16_	[M + H]+	Quercetin-3-O-(6′′-galloyl) glucoside	Flavonols	56316-75-7
Lmmp003903	491	287	490	C_23_H_22_O_12_	[M + H]+	Kaempferol-3-O-(2′′-acetyl) glucoside	Flavonols	–
Lmyp003873	617	153	616	C_28_H_24_O_16_	[M + H]+	Quercetin-3-O-(2′′-galloyl) glucoside	Flavonols	69624-79-9
mws0054	289	245	290	C_15_H_14_O_6_	[M − H]−	Catechin	Flavanols	154-23-4
pmn001526	483	169	484	C_20_H_20_O_14_	[M − H]−	1,6-Di-O-galloyl-D-glucose	Tannin	–
pmn001535	787	465	788	C_34_H_28_O_22_	[M − H]−	1,3,4,6-tetra-O-galloyl-D-glucose	Tannin	–
**R**								
Smsp002643	579	271	579	C_27_H_31_O_14_ +	[M]+	Pelargonidin-3-O-rutinoside	Anthocyanins	–
pme0434	577	407	578	C_30_H_26_O_12_	[M − H]−	Procyanidin B2	Proanthocyanidins	29106-49-8
pme0436	577	407	578	C_30_H_26_O_12_	[M − H]−	Procyanidin B3	Proanthocyanidins	23567-23-9
pmn001646	865	577	866	C_45_H_38_O_18_	[M − H]−	Procyanidin C1	Proanthocyanidins	37064-30-5
**L**								
Smlp002532	419	287	419	C_20_H_19_O_10_ +	[M]+	Cyanidin-3-O-arabinoside	Anthocyanins	27214-72-8
mws0072	433	271	432	C_21_H_20_O_10_	[M + H]+	Apigenin-5-O-glucoside	Flavonoid	28757-27-9
Lmpp003268	757	287	756	C_33_H_40_O_20_	[M + H]+	Kaempferol-3-O-rutinoside-7-O-glucoside	Flavonols	–
Lmmp001965	789	465	788	C_33_H_40_O_22_	[M + H]+	Gossypetin-3-O-rutinoside-8-O-glucoside	Flavonols	–
**Se**								
Lmyp004052	611	147	610	C_30_H_26_O_14_	[M + H]+	Quercetin-3-O-(6′′-p-coumaroyl) galactoside	Flavonols	–

*F, L, St, R, and Se represent the flower, leaves, stems, roots, and seeds of Hibiseu manihot L., respectively.*

### Analysis of the Differential Accumulated Flavonoids in Organs of *Hibiseu manihot* L.

We conducted further analysis of the differential accumulated flavonoids (DAFs) in different comparison groups (Supplementary Figure 2). A total of 144 DAFs were found and identified in JHK. Among them, 20 DAFs were downregulated and 82 DAFs were significantly accumulated in the L vs. F comparison group. In the R vs. F comparison group, 5 DAFs were downregulated and 114 DAFs were significantly accumulated. In the Se vs. F comparison group, only 1 DAF was downregulated and 121 DAFs were significantly accumulated. In the St vs. F comparison group, 6 DAFs were downregulated and 92 DAFs were significantly accumulated. The results showed that most of the DAFs were significantly accumulated in flowers of JHK, which was consistent with the results of PCA and total flavonoids detection.

**FIGURE 4:**
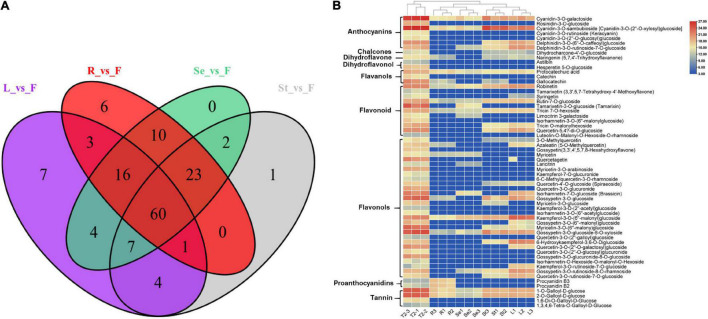
Differential accumulation of flavonoid metabolites analysis in different tissue parts of *Hibiseu manihot* L. Venn diagram depicting the shared and the specific number of flavonoids in different comparison groups **(A)**; the accumulation patterns of shared 60 flavonoid metabolites in different tissues of *Hibiseu manihot* L. **(B)**. In the above heat map, the red color indicates significant flavonoids accumulation, and the blue color indicates a significant reduction in flavonoids content. In LC-MS/MS profile analysis, F, L, St, R, and Se represent the flower, leaves, stems, roots, and seeds of *Hibiseu manihot* L., respectively.

We then performed a Venn analysis for the accumulation of DAFs in these comparison groups of JHK. The results showed that there was a total of 60 DAFs in different comparison groups (Figure 4A), which included flavonols (29), flavonoids (11), anthocyanins (7), and tannin (4). Figure 4B shows the content and accumulation of 60 common DAFs in different tissue parts of JHK. The results showed that most of these 60 DAFs were significantly accumulated in the flowers and partly in the stems as well. Compared with other tissue, two proanthocyanidins (procyanidin B2 and procyanidin B3) were only found significantly accumulated in the roots, whereas no specific significant accumulation DAFs were found in the seeds. Then we analyzed the specific (with high content) of DAFs in each component in different parts of JHK. Thirty-three flavonoids were identified with high concentrations only in the flowers, while these DAFs were not or at extremely low concentrations in leaves, roots, stems, and seeds (Table 1). These 33 DAFs were mainly including flavonols, flavonoid, and anthocyanins, such as hesperetin 5-O-glucoside, keracyanin, tamarixin, laricitrin, quercetagetin, etc. were only present in flowers of HJK. There were four unique accumulation DAFs in roots and leaves, respectively. Pelargonidin-3-O-rutinoside in roots, cyanidin-3-O-arabinoside, apigenin-5-O-glucoside, kaempferol-3-O-rutinoside-7-O-glucoside in leaves. And only one specific accumulation of DAFs in seeds [quercetin-3-O-(6′′-p-coumaroyl) galactoside]. However, no specific accumulation of DAFs was found in stems (Table 1). These results indicated that the specific accumulation and distribution pattern of these DAFs was the main reason for the difference of flavonoids in different tissue parts of JHK. Therefore, to further study the mechanism of changes in flavonoids at different tissue parts of JHK, we conducted transcriptome sequencing (RNA-seq) on these groups to further explore the possible pathways/mechanism that causes flavonoid accumulation differences in JHK.

### Transcriptome Sequencing (RNA-Seq) Analysis

Transcriptome sequencing was performed on 15 samples from different tissue parts of JHK. A total of 97.64 Gb of clean data was obtained through sequencing QC, and the clean data of each sample reached 5.81 Gb. The GC content was 42.98–44.84%, and the percentage of Q30 bases was >92.73% (Table 2). The results showed that the RNA-Seq profile was reliable. A total of 52,990 unigenes were obtained after assembling clean reads, among which 17,417 unigenes with lengths over 1 kb were obtained. Then we annotated the functions of these unigenes, including comparisons with NR, Swiss-Prot, KEGG, COG, KOG, GO, and PFAM databases, and obtained 39,651 unigenes annotation results. The annotated unigenes were analyzed for significant enrichment among different comparison groups, and the correlation analysis was carried out in combination with metabolomics (LC-MS/MS).

**TABLE 2:** Statistics, quality and RNA-Seq assembly results of 15 RNA sequencing libraries in different *Hibiseu manihot* L. tissues.

Sample name	Read number	Base number	GC content (%)	% ≥Q30 (%)	Clean reads	Mapped reads	Mapped ratio (%)
F1	19,524,709	5,812,427,242	44.22	92.73	19,524,709	13,871,475	71.05
F2	21,525,751	6,396,284,876	43.78	93.22	21,525,751	15,628,686	72.60
F3	22,185,555	6,632,429,268	44.09	92.86	22,185,555	16,248,610	73.24
L1	21,222,983	6,353,072,250	44.84	94.56	21,222,983	16,450,184	77.51
L2	21,609,587	6,461,562,668	44.56	94.29	21,609,587	16,449,980	76.12
L3	21,394,617	6,396,296,864	44.46	94.81	21,394,617	16,283,697	76.11
R1	21,349,514	6,389,722,464	45.34	94.24	21,349,514	15,905,201	74.50
R2	20,575,064	6,162,712,572	44.53	93.74	20,575,064	15,337,992	74.55
R3	35,829,521	10,670,717,516	44.73	95.02	35,829,521	26,593,132	74.22
Se1	21,156,288	6,156,076,676	44.57	94.11	21,156,288	13,168,409	62.24
Se2	20,809,092	6,128,271,216	43.88	93.89	20,809,092	13,794,102	66.29
Se3	21,112,487	6,119,196,198	44.81	94.09	21,112,487	12,710,695	60.20
St1	21,185,834	6,324,996,968	43.80	93.62	21,185,834	15,030,404	70.95
St2	19,558,292	5,805,616,484	43.01	93.15	19,558,292	13,641,579	69.75
St3	19,735,623	5,831,171,796	42.98	93.77	19,735,623	13,222,466	67.00

*F, L, St, R, and Se represent the flower, leaves, stems, roots, and seeds of Hibiseu manihot L., respectively. Read number: total number of pair-end reads in Clean Data. Base number: total base number of Clean Data.*

### Combined Transcriptome and Metabolome Analysis of *Hibiseu manihot* L.

Combined analysis of RNA-Seq and LC-MS/MS profiles were conducted by Pearson correlation analysis. The DEGs and related metabolites in the flavonoid biosynthesis pathway were identified in JHK. The expression level of 25 key genes in the phenylpropanoid (flavonoid) biosynthesis and flavone and flavonol biosynthesis pathways were significantly different in F, L, St, R, and Se of JHK (Supplementary Figure 3). Then we furtherly analyzed the genes and related metabolites significantly enriched in these pathways.

**FIGURE 5:**
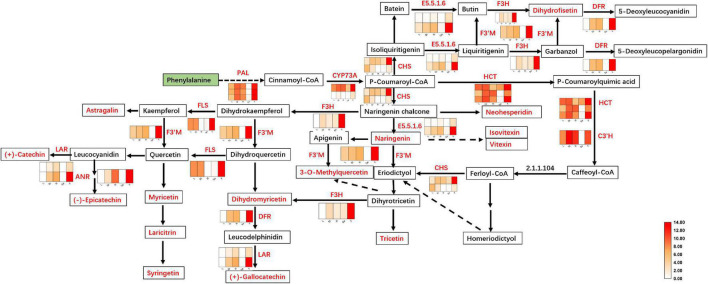
Flavonoids biosynthesis pathway in *Hibiseu manihot* L. The heat map in the above pathway represents the expression level of differentially expressed genes (DEGs). The boxes represent metabolites, and the red color indicates significantly accumulated flavonoids in *Hibiseu manihot* L. In the above heat map, the red color indicates the expression of genes significantly up-regulated, and the white color indicates the expression of genes significantly down-regulated. In each figure, F, L, St, R, and Se represent the flower, leaves, stems, roots, and seeds of *Hibiseu manihot* L., respectively.

**FIGURE 6:**
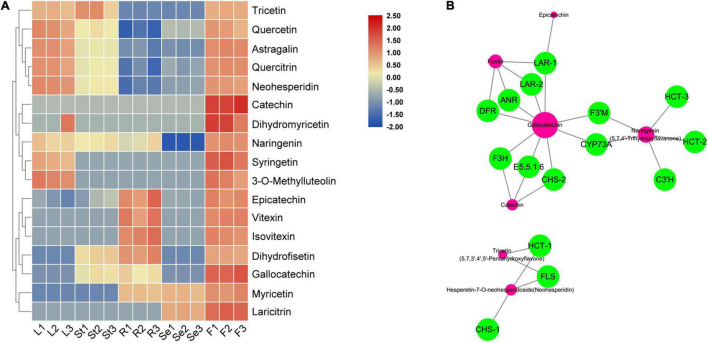
The heat map of significantly accumulated flavonoids that are enriched in flavonoid biosynthesis pathway **(A)** and transcriptome and metabolome combined analysis network **(B)** in *Hibiseu manihot* L. In the above heat map, the red color indicates flavonoids accumulation significantly, and the blue color indicates flavonoids content significant reduction. In the network, the green circles represent the DEGs and the red circles represent the corresponding metabolites. In each figure, F, L, St, R, and Se represent the flower, leaves, stems, roots, and seeds of *Hibiseu manihot* L., respectively.

Two of these genes (1 C3′H and 1 HCT) were downregulated in the F of JHK as compared to R and St, respectively. The expression level of 6 genes (LAR, DFR, ANR, CYP73A, and E5.5.1.6) did not shown any difference in L and Se. As a result, two coding genes of LAR were identified with no significant difference in expression between L and Se, while DFR, ANR, CYP73A and E5.5.1.6 were identified with only one coding gene with no significant difference between L and Se (Figure 5). However, 23 key genes were significantly upregulated in F as compared with L, St, R, and Se of JHK, respectively. These genes were involved in flavonoids synthesis in JHK. In addition, three phenylalanine ammonia-lyase (PAL) genes (c96791.graph_c0, c103354.graph_c0, and c79103.graph_c1) were found in our transcriptome profile, and they were all significantly upregulated in F. In JHK, PAL converts phenylalanine to cinnamoyl-CoA, which is then converted to p-coumaroyl-CoA by CYP73A enzyme catalysis. After some enzymes (CHS, E5.5.1.6, DFR, ANR, HCT, etc.) catalyzed the final synthesis of flavonoid compounds (Figure 5), the results of differentially accumulated flavonoids were consistent with the associated gene expression results (Figure 6A). All the 17 DAFs significantly enriched in flavonoids synthesis pathway were accumulated in F compared with L, Se, R, and St of JHK.

Among them, seven flavonoids were significantly associated with key enzyme genes of flavonoid synthesis, and their contents were regulated by the expression of these enzyme genes. Therefore, the above key genes have been used to co-express the network and character the genes that regulate metabolites (flavonoids) compounds. The genes, including *LAR*, *ANR*, *DFR*, *F3H*, *CHS*, *E5.5.1.6*, *F3*′*H*, *CYP73A*, *HCT*, *C3*′*H*, and *FLS*, have the highest degree of connectivity with gallocatechin, naringenin, dihydrofisetin (fustin), catechin, tricetin, epicatechin, and hesperetin-7-O-neohesperidoside (neohesperidin) biosynthesis (Figure 6B). Two hub metabolites (gallocatechin and naringenin) were significantly associated with key enzyme genes in flavonoid synthesis. The expression level of HCT, C3′H, DFR, F3H, FLS, CHS, and ANR genes were significantly related to the synthesis of flavonols (5), dihydroflavonol (4), flavonoid (3), flavanols (3), and flavonoid carbonoside (2) in JHK. Therefore, these results suggested that these structure genes might be a key regulator of flavonoids biosynthesis in JHK.

### Quantitative Real-Time PCR Verification

To verify the reliability of transcriptome for gene expression detection results, we not only used metabolite detection to verify the RNA-Seq results, but also carried out qRT-PCR detection for 20 key genes identified in this study (Supplementary Figure 4). These genes mainly include chalcone synthase (CHS), trans-cinnamate 4-monooxygenase (CYP73A), chalcone isomerase (CHI), naringenin 3-dioxygenase (F3H), bifunctional dihydroflavonol 4-reductase/flavanone 4-reductase (DFR), flavonoid 3′-monooxygenase (CYP75B1), shikimate O-hydroxycinnamoyltransferase (HCT), 5-O-(4-coumaroyl) -D-quinate 3′-monooxygenase (C3′H), leucoanthocyanidin reductase (LAR), anthocyanidin reductase (ANR), PAL, peroxidase (POD), caffeic acid 3-O-methyltransferase, cytochrome P450, and UDP-glycosyltransferase. The results showed that the trend of gene expression detected by qRT-PCR was consistent with the results of RNA-Seq data (*r*^2^ = 0.6213, *p* < 0.01). These key genes were all involved in the flavonoid biosynthesis in JHK, and might be used as candidate genes for further cloning and gene functional verification.

## Discussion

Jinhuakui is directly edible, and used in medicinal and health care functions for developing functional foods/products to improve humans health. It can also be used as garden plants having ornamental value. Flavonoids, an active medicinal substance, are the highest in JHK than other known natural products species (Lan et al., 2012; Li et al., 2012). Studies found that the extractive compounds from the JHK have been shown to decompose or scavenge DPPH, ABTS radical, hydroxyl, and superoxide radicals, inhibiting LDL oxidation (Yang et al., 2006) and chemo-preventive activities, and inhibiting xanthine-oxidase and lipid peroxidation (Wright et al., 2007). The flowers and calyces of JHK can be used to treat heart and nerve diseases, anti-tumor, diuretic, anti-scorbutic, sedative, colorectal, and intestinal antiseptic (Yang et al., 2006; Wright et al., 2007). The leaves and seeds of JHK were also used to treat conjunctivitis, ringworms, tumor, and abscesses or to alleviate headache, rheumatism, and hemorrhoid (Yang et al., 2006; Wright et al., 2007). JHK has high medicinal value and application prospects. Considering that JHK has so many pharmacological activities, it is of great significance to study the composition and synthesis mechanism of secondary metabolites in JHK.

Plant flavonoids have been illustrated to be antioxidants and anti-inflammatory against cerebral ischemia injury (Chen et al., 2020). They also have antiradical properties to prevent the associated diseases (Rice-Evans et al., 1996; Pan F. et al., 2017) by activating related gene expression, by interacting with MAP-kinase and PI3-kinase, and having an enzyme activity that can mediate cellular signaling transduction and other pathways (Seifried et al., 2007; Vauzour et al., 2010). Flavonoids were one of the main active metabolites in the JHK, and the accumulation of flavonoids in the flower can be up to 5.6% of the dry weight (Yang, 2013), which is ten times higher than that of ginkgo biloba and soybean (commonly used in industry to extract flavonoids) (Zhou et al., 2021). To systematically study the distribution and accumulation pattern of flavonoids in different organs of JHK, we combined metabolome (LC-MS/MS) with transcriptome sequencing to explore the flavonoids compound biosynthesis mechanism in different tissues of JHK. In the present study, we determined the total flavonoids content in JHK, and the results showed that the total flavonoids content in flowers was significantly higher than that in leaves, stems, and roots. Numerous studies have indicated a positive correlation between the contents of flavonoids and phenolic compounds and the antioxidant capacities in plant extracts (Aryal et al., 2019; Muflihah et al., 2021). All of the flavonoids in JHK had an efficient inhibitory effect on ROS production (Tsumbu et al., 2012) to treat certain diseases caused by the accumulation of ROS. Therefore, to make better use of the JHK plant, we recommend using the flower of this plant as a raw material to extract total flavonoids, which can improve the efficiency of flavonoids’ industrial production.

Although JHK has a high content of flavonoids, little information is available about the types and accumulation patterns of flavonoids in different tissues. In this study, a total of 160 flavonoids were identified in JHK by LC-MS/MS, and these flavonoids were mainly divided into 11 categories, of which the largest proportion was flavonols, followed by flavonoids and flavanols. At the same time, we found that the results of this experiment were similar to the results of the classification of flavonoids in *Abelmoschus Manihot* (relative species of JHK) (Pan X. et al., 2017), while there might be significant differences due to the content of these metabolites. Previous studies reported and identified eight major flavonoids in the flower of JHK (Li et al., 2019; Wu et al., 2019), which included vitexin rhamnoside, apigenin-8-C-glucoside (vitexin), rutin, hyperoside, quercetin, quercetin 3-O-robinobioside, quercetin-3-O-glucoside (isoquercitrin), and myricetin. Our results were consistent with these 8 kinds of flavonoids detected and identified by predecessors, and these DAFs have neuroprotective (Magalingam et al., 2014), antitumor (Huang et al., 2014; Lü, 2016), antioxidant, and anti-inflammatory effects (Ahn and Lee, 2016) by suppressing the activation of nuclear factor-κB in mouse peritoneal macrophages (Kim et al., 2011). In addition, the results of this study showed that spiraeoside, quercetin-3-O-glucuronide, quercetin-3-O-(2′′-O-glucosyl) glucuronide, quercetin-3-O-(6′′-galloyl) glucoside, and quercetin-3-O-(2′′-galloyl) glucoside were only determined in the flowers of JHK with high content, which have been illustrated with effects of antibacteria, anticonvulsant, antitumor, and anti-inflammatory activities (Chua, 2013) to inhibit partially the exocytosis of elastase and which might regulate the activation certain pathways (Selloum et al., 2003). Pelargonidin-3-O-rutinoside, apigenin-5-O-glucoside, and quercetin-3-O-(6′′-p-coumaroyl) galactoside were unique in the roots, leaves, and seeds of JHK, respectively.

In JHK, the accumulation of flavonoids was significantly associated with the flavonoid biosynthesis and the flavonol biosynthesis pathways, which directly target hundreds of flavonoid biosynthesis genes. There were significant differences in the accumulation patterns of flavonoids in different tissues/organs of JHK, and the related key genes that were significantly enriched in the pathway were also differentially expressed. Therefore, a significant cascade of transcriptional reprogramming and metabolite synthesis flow of flavonoids biosynthesis were studied in JHK. In the present study, we found that PAL catalyzes the conversion of phenylalanine to cinnamoyl-CoA, and then the CYP73A enzyme catalyzes the isomerization of cinnamoyl-CoA to p-coumaroyl-CoA. Compared with L, R, St, and Se, the genes coding PAL and CYP73A were significantly upregulated in F of JHK. The expression of these genes and upstream metabolic compounds accumulation pave the way for the synthesis of downstream flavonoids in JHK. CHI catalyzes the isomerization of naringenin chalcone to naringenin, which is a common precursor for the synthesis of different classes of flavonoids in plants (Shih et al., 2008), such as flavonols [3-O-methylquercetin, kaempferol-3-O-glucoside (astragalin), myricetin, laricitrin], flavonoid [tricetin (5, 7, 3′, 4′, 5′-pentahydroxyflavone), apigenin-6-C-glucoside (isovitexin), apigenin-8-C-glucoside (vitexin), syringetin, fustin (dihydrofisetin)], flavanols (gallocatechin, catechin, epicatechin), flavones [hesperetin-7-O-neohesperidoside (neohesperidin), dihydromyricetin (ampelopsin)], and anthocyanins [cyanidin-3-O-rutinoside (keracyanin), pelargonidin-3-O-rutinoside, petunidin-3-O-(6′′-O-p-coumaroyl) rutinoside-5-O-glucoside] identified in the present study. These DAFs have been illustrated with strong antioxidant and antiinflammation activities regulating certain signal transducers to alleviate related diseases (Cai et al., 2020; Zhao et al., 2020; Kim et al., 2021).

Myricetin, a dietary flavonoid, was identified as an active constituent in many herbal medicines, such as *Carthamus tinctorius* L. (Jin et al., 2003) and *Abelmoschus manihot* (Linn.) (Pan X. et al., 2017). Consistent with the previous studies, we identified five myricetin and their derivatives in JHK [myricetin, myricetin-3-O-(6′′-malony) glucoside, myricetin-3-O-arabinoside, myricetin-3-O-galactoside, myricetin-3-O-glucoside], which were active constituents to inhibit platelet aggregation (Survay et al., 2011) having anti-inflammatory, anticancer activities (Feng et al., 2015), and as natural B GPCR agonist or α-glucosidase inhibitor for type 2 diabetes treatment (Kang et al., 2015; Li et al., 2017). We found that myricetin-3-O-arabinoside was unique in the flowers of the JHK.

In the present study, we also identified the existence of other kinds of flavonoids having high content that was not identified and reported in JHK before, such as catechin, cyanidin-3-O-sambubioside [cyanidin-3-O-(2′′-O-xylosyl) glucoside], luteolin-4′-O-β-D-glucoside, kaempferol-3-O-glucoside (astragalin), procyanidin B2/B3/C1, chrysoeriol-7-O-glucoside, apigenin-5-O-glucoside, hesperetin-7-O-rutinoside (hesperidin), etc. These flavonoids identified in this study have been reported for the first time in JHK. Three hesperetin (hesperetin 5-O-glucoside, neohesperidin, hesperetin-7-O-rutinoside) and bioactive flavonoid (vitamin P) were identified with a high concentration in JHK that has been well demonstrated for the medicinal properties as an important Chinese traditional medicine (Formica and Regelson, 1995), and which has also been used in cosmetics due to its anti-allergic and antioxidant activities (Fujitaka et al., 2019). Among them, hesperetin 5-O-glucoside was only determined to exist in the flowers of JHK. Studies have found that hesperetin with antioxidant and antiallergic activities regulates certain signal transduction pathways, blood-brain barrier, and treats tumors (O’Prey et al., 2003; Youdim et al., 2003). Although other flavonoid compounds have not yet been reported with pharmacological functions, they were found in high concentrations in JHK by LC-MS/MS in the present study. Therefore, these compounds can be isolated and further studied to develop their medicinal function for human health benefits.

## Data Availability Statement

The original contributions presented in the study are publicly available. This data can be found here: National Center for Biotechnology Information (NCBI) BioProject database under accession number PRJNA786451.

## Author Contributions

LC: conceptualization, funding acquisition, project administration, resources, and writing—review and editing. YZ: data curation, formal analysis, investigation, methodology, software, and roles/writing—original draft. XX: formal analysis and writing—review and editing. YC: software and writing—review and editing. JG: validation and writing—review and editing. QS and LT: writing—review and editing. All authors contributed to the article and approved the submitted version.

## Conflict of Interest

The authors declare that the research was conducted in the absence of any commercial or financial relationships that could be construed as a potential conflict of interest.

## Publisher’s Note

All claims expressed in this article are solely those of the authors and do not necessarily represent those of their affiliated organizations, or those of the publisher, the editors and the reviewers. Any product that may be evaluated in this article, or claim that may be made by its manufacturer, is not guaranteed or endorsed by the publisher.

## Footnotes

1
https://www.shimadzu.com.cn/

2
http://www.appliedbiosystems.com.cn/

3
www.metware.cn

4
https://data.broadinstitute.org/chembank/assay/index.html

5
https://pubchemblog.ncbi.nlm.nih.gov/

6
http://www.massbank.jp/

7
http://webbook.nist.gov/

8
http://kanaya.naist.jp/KNApSAcK/

9
http://appliedbioinformatics.wur.nl

10
http://www.hmdb.ca/

11
http://metlin.scripps.edu/index.php

12
www.r-project.org

13
www.biocloud.net

14
https://bioinfo.ut.ee/primer3-0.4.0/

15
http://cran.r-project.org/

## Abbreviations

JHK: *Hibiseu manihot* L.
RNA-seq: transcriptomics
MRM: multiple reaction monitoring
OPLS-DA: orthogonal partial least squares-discriminant analysis
PCA: principal component analysis
VIP: variable importance in projection
DAMs: differentially accumulated metabolites
qRT-PCR: quantitative real-time PCR
QC: quality control
TIC: total ion chromatogram
PAL: phenylalanine ammonia-lyase
CHS: chalcone synthase
CYP73A: trans-cinnamate 4-monooxygenase
CHI: chalcone isomerase
F3H: naringenin 3-dioxygenase
DFR: bifunctional dihydroflavonol 4-reductase/flavanone 4-reductase
CYP75B1: flavonoid 3′-monooxygenase
HCT: shikimate O-hydroxycinnamoyltransferase
C3′H: 5-O-(4-coumaroyl-D-quinate 3′-monooxygenase
LAR: leucoanthocyanidin reductase
ANR: anthocyanidin reductase
POD: peroxidase.

## References

[B1] Ahn H. Lee G. S. (2016). Isorhamnetin and hyperoside derived from water dropwort inhibits inflammasome activation. *Phytomedicine* 24 77–86. 10.1016/j.phymed.2016.11.019 28160865

[B2] Aryal S. Baniya M. K. Danekhu K. Kunwar P. Gurung R. Koirala N. (2019). Total phenolic content, flavonoid content and antioxidant potential of wild vegetables from Western Nepal. *Plants* 8:96. 10.3390/plants8040096 30978964 PMC6524357

[B3] Cai L. Zhang X. Hou M. Gao F. (2020). Natural flavone tricetin suppresses oxidized LDL-induced endothelial inflammation mediated by Egr-1. *Int. Immunopharmacol.* 80:106224. 10.1016/j.intimp.2020.106224 31991371

[B4] Cao L. H. Miao M. S. (2016). Analysis of modern research and comprehensive utilization of Aurea *Helianthus*. *Acta Chin. Med.* 31 1966–1968.

[B5] Chen J. Yang J. Ma L. Li J. Shahzad N. Kim C. K. (2020). Structure-antioxidant activity relationship of methoxy, phenolic hydroxyl, and carboxylic acid groups of phenolic acids. *Sci. Rep.* 10:2611. 10.1038/s41598-020-59451-z 32054964 PMC7018807

[B6] Chen L. M. Dong S. Liang W. M. Yang L. L. Chen Y. (2016). Researches on extraction and antioxidant activity of soluble carbohydrate from stalk of Aurea *Helianthus*. *Food Res. Dev.* 37 59–63.

[B7] Chen L. Xin X. L. Su D. H. Wang X. J. Hu X. M. Li B. (2016). Study on refining technology of total flavonoids from *Hibiseu smanihot* L. *J. Jiangsu Agric. Sci.* 44 339–343.

[B8] Chua L. S. (2013). A review on plant-based rutin extraction methods and its pharmacological activities. *J. Ethnopharmacol.* 150 805–817. 10.1016/j.jep.2013.10.036 24184193

[B9] Cui Q. Liu J. Z. Yu L. Gao M. Z. Wang L. T. Wang W. (2020). Experimental and simulative studies on the implications of natural and green surfactant for extracting flavonoids. *J. Clean. Prod.* 274 122652.

[B10] Fan H. T. Wei W. B. Wang Y. Du C. S. Huang J. M. Zheng Z. X. (2020). Differences of different cultivation methods and different densities of *Hibiseu manibot* L. *Northern Hortic.* 20 70–76.

[B11] Feng J. F. Chen X. N. Wang Y. Y. Du Y. W. Sun Q. Q. Zang W. Q. (2015). Myricetin inhibits proliferation and induces apoptosis and cell cycle arrest in gastric cancer cells. *Mol. Cell. Biochem.* 408 163–170. 10.1007/s11010-015-2492-1 26112905

[B12] Formica J. V. Regelson W. (1995). Review of the biology of quercetin and related bioflavonoids. *Food Chem. Toxicol.* 33 1061–1080. 10.1016/0278-6915(95)00077-1 8847003

[B13] Fraga C. G. Clowers B. H. Moore R. J. Zink E. M. (2010). Signature-discovery approach for sample matching of a nerve-agent precursor using liquid chromatography-mass spectrometry, XCMS, and chemometrics. *Anal. Chem.* 82 4165–4173. 10.1021/ac1003568 20405949

[B14] Fujitaka Y. Hamada H. Uesugi D. Kuboki A. Shimoda K. Iwaki T. (2019). Synthesis of daidzein glycosides, α-tocopherol glycosides, hesperetin glycosides by bioconversion and their potential for anti-allergic functional-foods and cosmetics. *Molecules* 24:2975. 10.3390/molecules24162975 31426346 PMC6721765

[B15] Huang G. Tang B. Tang K. Dong X. Deng J. Liao L. (2014). Isoquercitrin inhibits the progression of liver cancer in vivo and in vitro via the MAPK signalling pathway. *Oncol. Rep.* 31 2377–2384. 10.3892/or.2014.3099 24676882

[B16] Jin M. Wang Y. Q. Li J. S. Wang X. K. (2003). Isolation and identification of flavonols from *Carthamus tinctorious* L. *Chin. Trad. Herb. Drugs* 34 306–308.

[B17] Kang S. J. Park J. H. Y. Choi H. N. Kim J. I. (2015). Alpha-glucosidase inhibitory activities of myricetin in animal models of diabetes mellitus. *Food Sci. Biotechnol.* 24 1897–1900. 10.1016/j.biopha.2021.111859 34246953

[B18] Kim S. J. Um J. Y. Lee J. Y. (2011). Anti-inflammatory activity of hyperoside through the suppression of nuclear factor-κB activation in mouse peritoneal macrophages. *Am. J. Chin. Med.* 39 171–181. 10.1142/S0192415X11008737 21213407

[B19] Kim Y. H. Kang M. K. Lee E. J. Kim D. Y. Oh H. Kim S. I. (2021). Astragalin inhibits cigarette smoke-induced pulmonary thrombosis and alveolar inflammation and disrupts PAR activation and oxidative stress-responsive MAPK-signaling. *Int. J. Mol. Sci.* 22:3692. 10.3390/ijms22073692 33916310 PMC8036420

[B20] Lai X. Liang H. Zhao Y. Wang B. (2009). Simultaneous determination of seven active flavonols in the flowers of *Abelmoschus manihot* by HPLC. *J. Chromatogr. Sci.* 3 206–210. 10.1093/chromsci/47.3.206 19298707

[B21] Lan R. Li B. Liu H. Chen L. (2012). Extraction and determination of total flavone in *Hibiseu manihot* L. *J. Jiangsu Agric. Sci.* 40 280–282. 10.15889/j.issn.1002-1302.2012.12.045

[B22] Li B. Hu D. Y. Li S. S. Xin X. L. Chen L. Liu Q. (2012). Determination of the unsaturated fatty acids of *Hibiscus manihot* seed oil by GC-MS. *Food Res. Dev.* 33 121–123.

[B23] Li C. Xin M. Li L. He X. Yi P. Tang Y. (2021). Characterization of the aromatic profile of purple passion fruit (*Passiflora edulis* Sims) during ripening by HS-SPME-GC/MS and RNA sequencing. *Food Chem.* 355:129685. 10.1016/j.foodchem.2021.129685 33799248

[B24] Li J. Wang Y. Rong J. Zhan S. Wang Z. (2019). Nutritional value and antioxidant activity of *Abelmoschus manihot*. *Chin. J. Trop. Crops* 40 1354–1358.

[B25] Li S. Deng B. Tian S. Guo M. Liu H. Zhao X. (2021). Metabolic and transcriptomic analyses reveal different metabolite biosynthesis profiles between leaf buds and mature leaves in *Ziziphus jujuba* mill. *Food Chem.* 347:129005. 10.1016/j.foodchem.2021.129005 33482487

[B26] Li Y. Kong D. Fu Y. Sussman M. R. Wu H. (2020). The effect of developmental and environmental factors on secondary metabolites in medicinal plants. *Plant Physiol. Biochem.* 148 80–89. 10.1016/j.plaphy.2020.01.006 31951944

[B27] Li Y. Zheng X. M. Yi X. L. Liu C. X. Kong D. X. Zhang J. N. (2017). Myricetin: a potent approach for the treatment of type 2 diabetes as a natural class B GPCR agonist. *FASEB J.* 31 2603–2611. 10.1096/fj.201601339R 28270518 PMC5434659

[B28] Liu L. (2008). Determination of trace element of *Abelmoschus manihot* by means of atom absorption spectra. *Stud. Trace Elements Health* 04 27–29.

[B29] Lu D. Jia R. B. (2015). Research progress on Chinese medicinal material Aurea *Helianthus*. *Chin. J. Drug Eval.* 32 90–92.

[B30] Lü P. (2016). Inhibitory effects of hyperoside on lung cancer by inducing apoptosis and suppressing inflammatory response via caspase-3 and NF-κB signaling pathway. *Biomed. Pharmacother.* 82 216–225. 10.1016/j.biopha.2016.05.006 27470358

[B31] Magalingam K. B. Radhakrishnan A. Haleagrahara N. (2014). Protective effects of flavonol isoquercitrin, against 6-hydroxy dopamine (6-OHDA)-induced toxicity in PC12 cells. *BMC Res. Notes* 7:49. 10.1186/1756-0500-7-49 24443837 PMC3910241

[B32] Morgenthal K. Weckwerth W. Steuer R. (2006). Metabolomic networks in plants: transitions from pattern recognition to biological interpretation. *Bio. Syst.* 83 108–117. 10.1016/j.biosystems.2005.05.017 16303239

[B33] Muflihah Y. M. Gollavelli G. Ling Y. C. (2021). Correlation study of antioxidant activity with phenolic and flavonoid compounds in 12 Indonesian indigenous herbs. *Antioxidants* 10:1530. 10.3390/antiox10101530 34679665 PMC8533117

[B34] O’Prey J. Brown J. Fleming J. Harrison P. R. (2003). Effects of dietary flavonoids on major signal transduction pathways in human epithelial cells. *Biochem. Pharm.* 66 2075–2088. 10.1016/j.bcp.2003.07.007 14609732

[B35] Pan F. Su T. J. Cai S. M. Wu W. (2017). Fungal endophyte-derived *Fritillaria unibracteata* var. wabuensis: diversity, antioxidant capacities in vitro and relations to phenolic, flavonoid or saponin compounds. *Sci. Rep.* 7:42008. 10.1038/srep42008 28165019 PMC5292746

[B36] Pan X. Du L. Tao J. Jiang S. Qian D. Duan J. (2017). Dynamic changes of flavonoids in *Abelmoschus manihot* different organs at different growth periods by UPLC-MS/MS. *J. Chromatogr. B Analyt. Technol. Biomed. Life Sci.* 1059 21–26. 10.1016/j.jchromb.2017.05.020 28558340

[B37] Peng Z. B. Wu Z. B. (2008). Analysis of hyperosidein flower of *Hibiscus manihot* L. by HPLC-mass spectrometry. *J. Anhui Agric. Sci.* 36 10028–10029.

[B38] Rice-Evans C. A. Miller N. J. Paganga G. (1996). Structure-antioxidant activity relationships of flavonoids and phenolic acids. *Free Radic. Biol. Med.* 20 933–956. 10.1016/0891-5849(95)02227-9 8743980

[B39] Seifried H. E. Anderson D. E. Fisher E. I. Milner J. A. (2007). A review of the interaction among dietary antioxidants and reactive oxygen species. *J. Nutr. Biochem.* 18 567–579. 10.1016/j.jnutbio.2006.10.007 17360173

[B40] Selloum L. Bouriche H. Tigrine C. Boudoukha C. (2003). Anti-inflammatory effect of rutin on rat paw oedema, and on neutrophils chemotaxis and degranulation. *Exp. Toxicol. Pathol.* 54 313–318. 10.1078/0940-2993-00260 12710715

[B41] Shih C. H. Chen Y. Wang M. Chu I. K. Lo C. (2008). Accumulation of isoflavone genistin in transgenic tomato plants overexpressing a soybean isoflavone synthase gene. *J. Agric. Food Chem.* 56 5655–5661. 10.1021/jf800423u 18540614

[B42] Song L. L. Huang B. B. Zhang C. H. (2016). Ultrasonic extraction technology of total flavone from flower of *Hibiscus manihot*. *Guizhou Agric. Sci.* 44 70–72.

[B43] Survay N. S. Upadhyaya C. P. Kumar B. Young K. E. Yoon D. Y. Park S. W. (2011). New genera of flavonols and flavonol derivatives as therapeutic molecules. *J. Korean Soc. Appl. Biol. Chem.* 54 1–18. 10.3390/molecules21010011 26703549 PMC6273011

[B44] Sytar O. Borankulova A. Hemmerich I. Rauh C. Smetanska I. (2014). Effect of chlorocholine chlorid on phenolic acids accumulation and polyphenols formation of buckwheat plants. *Biol. Res.* 47:19. 10.1186/0717-6287-47-19 25027783 PMC4101719

[B45] Sytar O. Cai Z. Brestic M. Kumar A. Prasad M. N. V. Taran N. (2013). Foliar applied nickel on buckwheat (fagopyrum esculentum) induced phenolic compounds as potential antioxidants. *CLEAN Soil Air Water* 41 1129–1137. 10.1002/clen.201200512

[B46] Tsumbu C. N. Deby-Dupont G. Tits M. Angenot L. Frederich M. Kohnen S. (2012). Polyphenol content and modulatory activities of some tropical dietary plant extracts on the oxidant activities of neutrophils and myeloperoxidase. *Int. J. Mol. Sci.* 13 628–650. 10.3390/ijms13010628 22312276 PMC3269710

[B47] Vauzour D. Rodriguez-Mateos A. Corona G. Oruna-Concha M. J. Spencer J. P. (2010). Polyphenols and human health: prevention of disease and mechanisms of action. *Nutrients* 2 1106–1131. 10.3390/nu2111106 22254000 PMC3257622

[B48] Wang M. Z. (2015). *The Extraction and Separation of Flavone in Hibiscus manihot Flower and the Investigation of Its Activities.* Hebei: Hebei University of Science and Technology.

[B49] Wang M. Kang X. Deng L. Wang M. Xia Z. Gao D. (2021). Deep eutectic solvent assisted synthesis of carbon dots using *Sophora flavescens* Aiton modified with polyethyleneimine: application in myricetin sensing and cell imaging. *Food Chem.* 30 128817. 10.1016/j.foodchem.2020.128817 33307432

[B50] Wang S. Liu L. Mi X. Zhao S. An Y. Xia X. (2021). Multi-omics analysis to visualize the dynamic roles of defense genes in the response of tea plants to gray blight. *Plant J.* 106 862–875. 10.1111/tpj.15203 33595875

[B51] Wei Q. Lan R. Xin X. L. Chen L. (2012). Determination of total flavonoids content in Golden Kwai seed by ultraviolet spectrophotometry. *J. Anhui Agric. Sci.* 40 7050–7060.

[B52] Wright C. I. Van-Buren L. Kroner C. I. Koning M. M. (2007). Herbal medicines as diuretics: a review of the scientific evidence. *J. Ethnopharmacol.* 114 1–31. 10.1016/j.jep.2007.07.023 17804183

[B53] Wu Z. Li L. Xu M. Zang J. (2019). Simultaneous determination of five flavonoids in the flowers of *Abelmoschus manihot* by high performance liquid chromatography. *Chin. Food Industry* 40 284–287.

[B54] Xia W. K. (2007). *Studies on Extraction and Antioxidant Activity of Total Flavones From Hibiscus manihot L. Flower.* Jingkou: Jiangsu University.

[B55] Yang R. Y. Tsou S. C. S. Lee T. C. Hanson W. J. Kuo P. M. (2006). Distribution of 127 edible plant species for antioxidant activities by two assays. *J. Sci. Food Agric.* 86 2395–2403.

[B56] Yang X. S. (2013). Immunoregulation of crude flavonoids from *Abelmoschus manihot* flower. *China Pharmacist* 16 1307–1311.

[B57] Yi D. Zhang H. Lai B. Liu L. Pan X. Ma Z. (2021). Integrative analysis of the coloring mechanism of red longan pericarp through metabolome and transcriptome analyses. *J. Agric. Food Chem.* 69 1806–1815. 10.1021/acs.jafc.0c05023 33332135

[B58] Youdim K. A. Dobbie M. S. Kuhnle G. Proteggente A. R. Abbott N. J. Rice-Evans C. (2003). Interaction between flavonoids and the blood-brain barrier: In vitro study. *J. Neurochem.* 85 180–192. 12641740 10.1046/j.1471-4159.2003.01652.x

[B59] Yu Z. Y. (2021). Effect of exogenous calcium on seed germination of Aurea *Helianthu* under salt stress. *Modern Chin. Hortic.* 44 53–54.

[B60] Zhang D. W. Ma K. Tian P. (2019). Simultaneous determination of 11 active components in *Aurea helianthus* by UPLC-MS/MS. *Chin. J. Pharm. Anal.* 39 780–786.

[B61] Zhao X. Chen R. Shi Y. Zhang X. Tian C. Xia D. (2020). Antioxidant and anti-inflammatory activities of six flavonoids from Smilax glabra Roxb. *Molecules* 25:5295. 10.3390/molecules25225295 33202848 PMC7697956

[B62] Zhou Y. H. Xu X. D. Shi Q. Y. Gao J. Cao L. (2021). Optimization of extraction of total flavonoids from *Hibiseu manihot* L. and study on its components. *Agric. Sci. J. Yanbian Univ.* 43 24–30.

[B63] Zhu Y. Wang Q. Wang Y. Xu Y. Li J. Zhao S. (2021). Combined transcriptomic and metabolomic analysis reveals the role of phenylpropanoid biosynthesis pathway in the salt tolerance process of *Sophora alopecuroides*. *Int. J. Mol. Sci.* 22:2399. 10.3390/ijms22052399 33673678 PMC7957753

