# Supplementary material for: Combined Metabolome and Transcriptome Analyses Reveal the Flavonoids Changes and Biosynthesis Mechanisms in Different Organs of Hibiseu manihot L
Source: Front Plant Sci. 2022 Mar 15;13:817378. doi: 10.3389/fpls.2022.817378 (PMC8965375; doi:10.3389/fpls.2022.817378)
Supplement: Supplementary file 1 [file Table_1.docx]

Table S1. Primer list used by qRT-PCR analysis.

| Gene-id |  | Sequences (5'-3') | Product length |
| --- | --- | --- | --- |
| AeActin | F | TCTTTCATCGGGATGGAAGC | 108bp |
|  | R | ACTGAGCACAATGTTACCGTAGAG |  |
| c102649.graph_c0 | F | ATCGGCACATCCACTCCAC | 145bp |
|  | R | TGTACCGCTTCTGAATCATGG |  |
| c96512.graph_c0 | F | GGCTATCAAAGAATGGGGTCA | 122bp |
|  | R | TGGGTCTCAGTCCAAGCAGTT |  |
| c79566.graph_c0 | F | GGATGCTGTCGTGCTATCTGG | 113bp |
|  | R | TGTTGAAGGTTGGAGCTGGTAAT |  |
| c102201.graph_c0 | F | CACAGTGTCGGCAGAACTCAC | 109bp |
|  | R | CAGGGCACTTGTGGAGCAT |  |
| c106683.graph_c0 | F | TTCGAAAGCGAGGACGATC | 126p |
|  | R | CAAGAAAGGTCTCAAAATGGG |  |
| c81818.graph_c0 | F | TACCTCACCGTCCATCACCC | 198bp |
|  | R | CACTTAGCGGCAAGGCAATC |  |
| c104820.graph_c0 | F | CATCGTCTCCAGCCACCTTC | 135bp |
|  | R | TTTGTCACTTCAATCCAACCCTC |  |
| c102557.graph_c1 | F | AGCTGATGGACTTGGGGTTT | 128bp |
|  | R | TTTTCCGTTCCCGTTGACA |  |
| c101539.graph_c0 | F | TGAAGGCGGAAGGTTTCTTT | 131bp |
|  | R | CATCCCACGATCATGGCTC |  |
| c101021.graph_c0 | F | ATCCCATACTTACCGATGTCCG | 189bp |
|  | R | GCTCCCAAGCGAGTTACCAGA |  |
| c111537.graph_c0 | F | GCTGAATCCTCGTCCTCCAC | 148bp |
|  | R | GTACTCGTCATCCACCTGCTGT |  |
| c93893.graph_c0 | F | GGACACTATCATTGGACTTCTTTGG | 162bp |
|  | R | AACATCACCCTTTCAGAACCTACC |  |
| c99979.graph_c0 | F | ATCGGTTCGAGCGGGTTT | 113bp |
|  | R | GTGGCTTTGGTTGAGGTAGGG |  |
| c104706.graph_c0 | F | TGACATGATAAAACCGGCAATC | 172bp |
|  | R | GAAGTCGGTATCGGTCCAATCT |  |
| c96791.graph_c0 | F | GCAGCACAACCAAGATGTAAACT | 162bp |
|  | R | TTCTTCACCGTGTTCCTCAAA |  |
| c103354.graph_c0 | F | CCCTGGGACTAATATCTTCACG | 191bp |
|  | R | TTCGCCATTGGCACCTGT |  |
| c79103.graph_c1 | F | CGGGTCTTTGGAGGGATTT | 122bp |
|  | R | CCACCATTCGTTTCACTTCG |  |
| c108135.graph_c0 | F | TTCCCCGAAAGAAGTGGC | 107bp |
|  | R | AGCAGGTGAGGATGGAGTAGC |  |
| c103515.graph_c0 | F | CTCGCCAAGTGCTTCAGGT | 124bp |
|  | R | TGTCTCCAAAATGGTCCGTAAT |  |
| c104309.graph_c0 | F | TTCCTTTGGGTGGTTCGTG | 107bp |
|  | R | AGAAACCCATCGGGCAAGT |  |
