# Supplementary material for: Combined Metabolome and Transcriptome Analyses Reveal the Flavonoids Changes and Biosynthesis Mechanisms in Different Organs of Hibiseu manihot L
Source: Front Plant Sci. 2022 Mar 15;13:817378. doi: 10.3389/fpls.2022.817378 (PMC8965375; doi:10.3389/fpls.2022.817378)
Supplement: Supplementary file 3 [file Data_Sheet_1.docx]

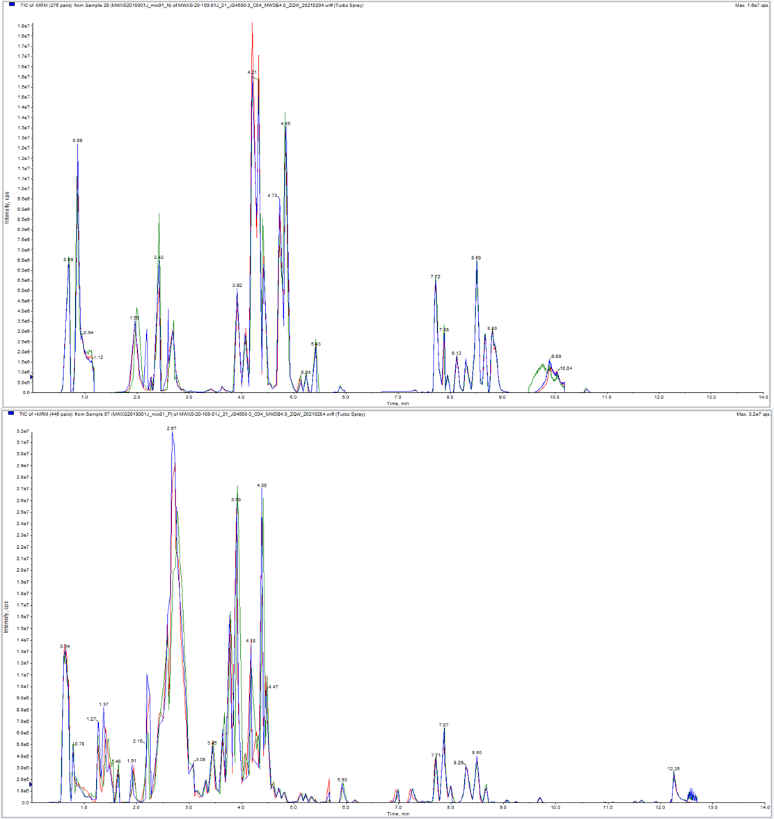


Figure S1 Overlap comparison of the total ion chromatogram (TIC) in quality control *Hibiseu manihot* L. samples.


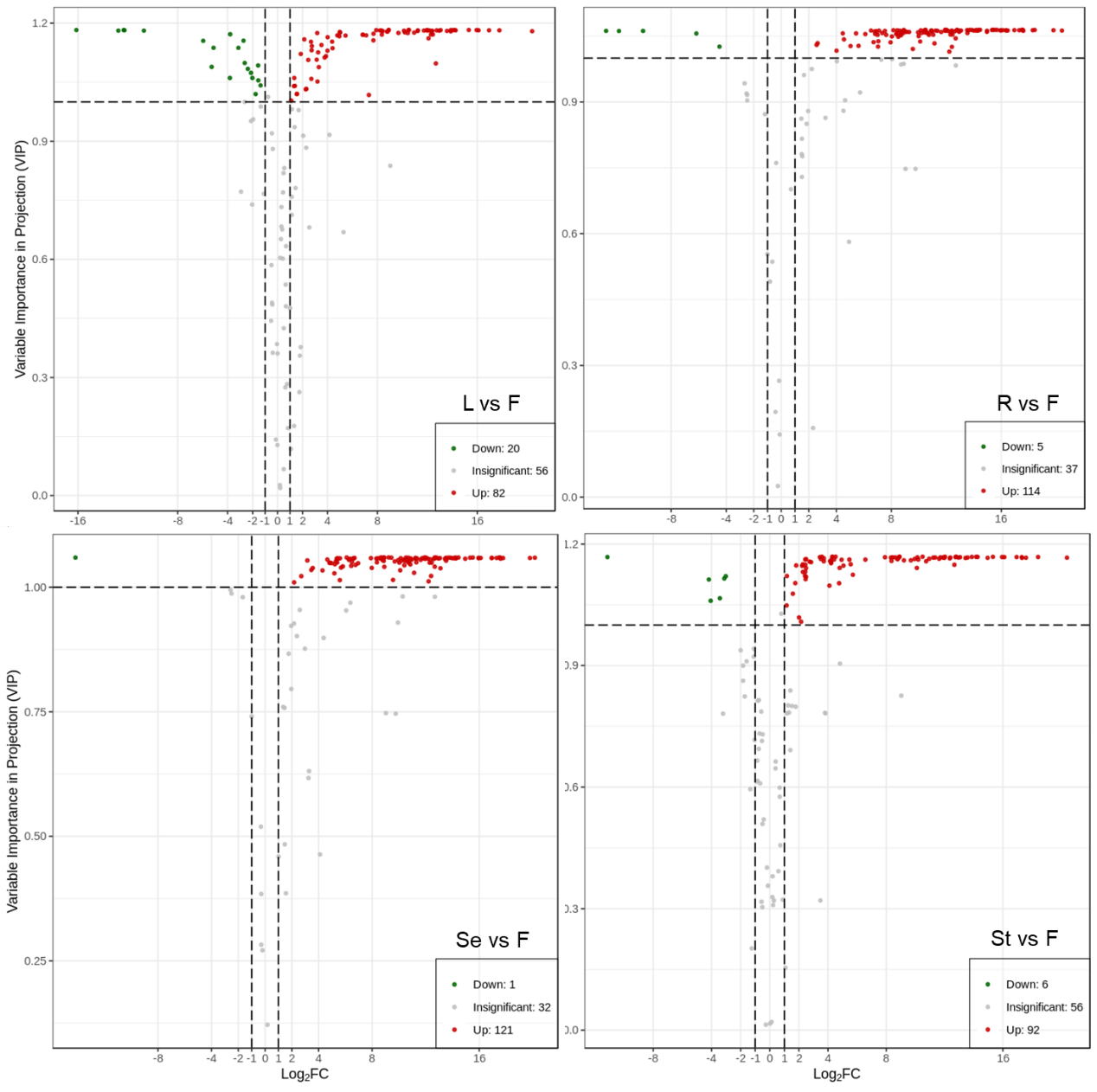


Figure S2 Volcano plots of differentially accumulated flavonoids (DAFs) with different comparison (L vs F, R vs F, Se vs F and St vs F) in *Hibiseu manihot* L. The green dots represent down-accumulated DAFs and the red dots represent up-accumulated DAFs between different caparisons. In LC-MS/MS profile analysis, F, L, St, R and Se represent the flower, leaves, stems, roots and seeds of Hibiseu manihot L., respectively.


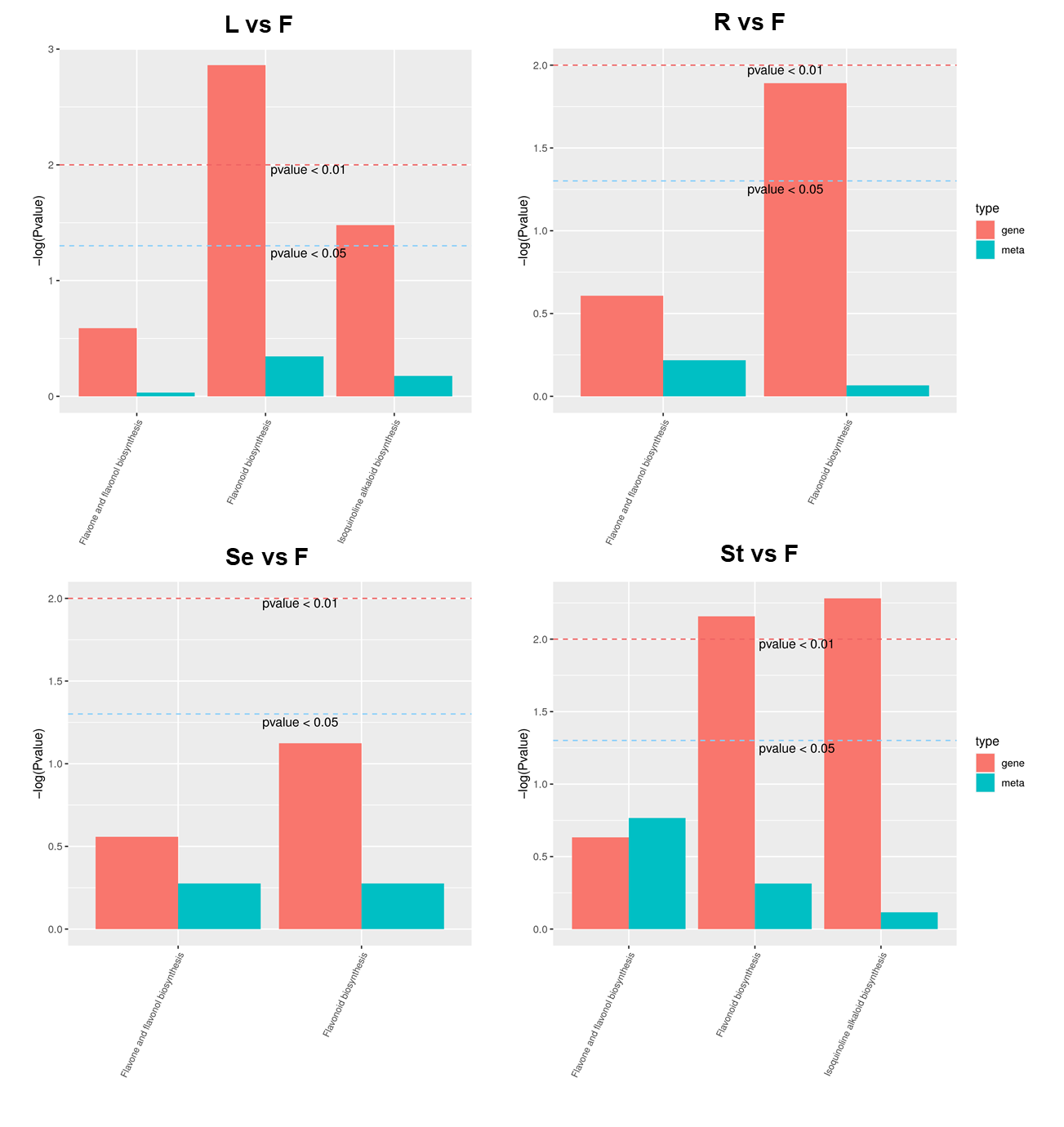


Figure S3 Combined transcriptome and metabolome analysis.Pathway enrichment of differentially accumulated flavonoids (DAFs) and differentially expressed genes (DEGs) in each comparison group of *Hibiseu manihot* L. In above figures, F, L, St, R and Se represent the flower, leaves, stems, roots and seeds of *Hibiseu manihot* L., respectively.


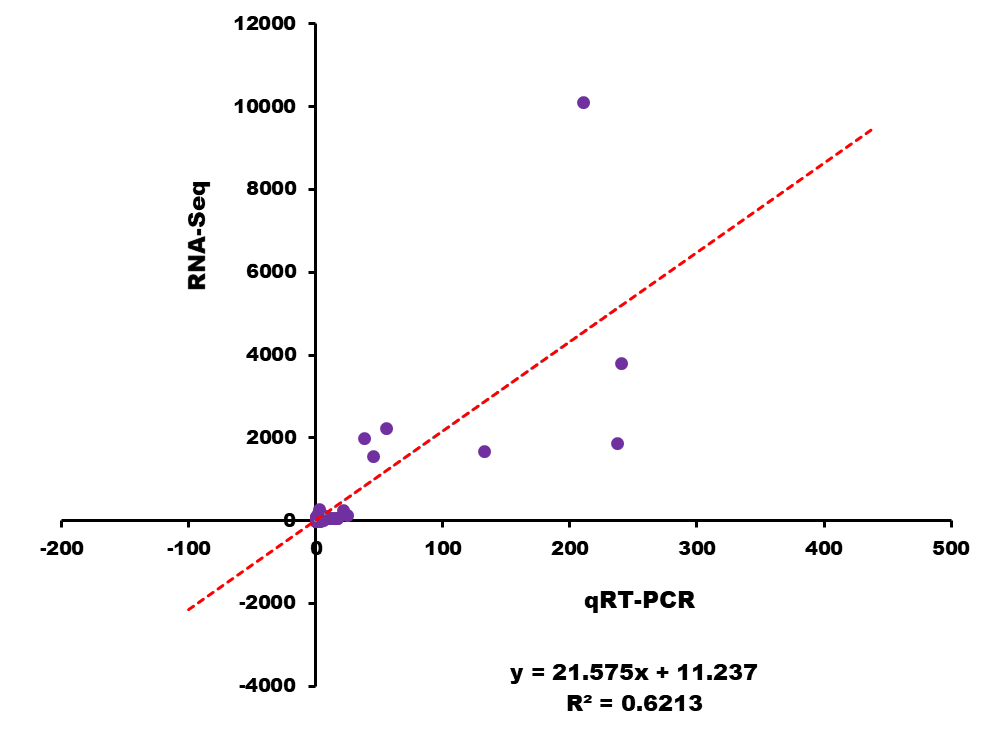


Figure S4 Correlations between qRT-PCR data and RNA-Seq profile for the 20 unigenes. Each point represents a fold change value of gene expression level in different tissues of *Hibiseu manihot* L.
